# Automated dialysate sodium control system: a word of caution regarding potassium

**DOI:** 10.1093/ckj/sfaf018

**Published:** 2025-01-28

**Authors:** Maxime Ingwiller

**Affiliations:** Nephrology Department, CHU de Strasbourg, Service de Néphrologie, CHU de Strasbourg, France

To the Editor,

Recent advancements in hemodialysis technology, particularly sodium control mechanisms such as those in the Fresenius 6008 generator, have shown promise in maintaining sodium balance during dialysis [1, 2]. However, these automated systems may have limitations for hyperkalemic patients, which I would like to highlight.

First, it is important to revisit the fundamental basis of these algorithms, designed to achieve a zero diffusive sodium balance [3].


(1) \begin{eqnarray*}
{\mathrm{Given\ that\ \Delta }}{{\mathrm{J}}_{{\mathrm{diff}}}}={{\mathrm{Q}}_{\mathrm{d}}}{\mathrm{*}}\left( {{\mathrm{C}}{{\mathrm{d}}_{\mathrm{i}}}{\mathrm{ - C}}{{\mathrm{d}}_{{\mathrm{out}}}}} \right)
\end{eqnarray*}

where ${\mathrm{\Delta }}{{\mathrm{J}}_{{\mathrm{diff}}}}$ is the mass balance, representing the total amount of solute transferred into or out of the patient through diffusion per unit time, and ${{\mathrm{Q}}_{\mathrm{d}}}$ is the dialysate flow rate. To achieve zero diffusive balance, the algorithm adjusts ${\mathrm{C}}{{\mathrm{d}}_{\mathrm{i}}}$ (dialysate sodium concentration) to match ${\mathrm{C}}{{\mathrm{d}}_{{\mathrm{out}}}}$ (spent dialysate concentration) until ${\mathrm{\Delta }}{{\mathrm{J}}_{{\mathrm{diff}}}}$ reaches zero.

Since dialysate conductivity is governed by the electrolyte concentration where sodium is the dominant cation, conductivity was used as a reliable proxy for sodium concentration in the dialysate. However, the initial version of the algorithm led to significant increases in mean plasma sodium levels, as it did not account for potassium change during treatment [3].

Potassium, the second most prevalent cation in the dialysate, generally has a negative balance during dialysis. Therefore, maintaining a zero-conductivity balance requires a positive sodium load to offset the potassium loss, keeping dialysate conductivity stable: an important factor initially overlooked, prompting algorithm correction [1].

The corrected sodium change (${\mathrm{\Delta N}}{{\mathrm{a}}_{{\mathrm{p}}}{{\mathrm{,}}_{{\mathrm{corr}}}}}$) can be calculated as follows:


(2) \begin{eqnarray*}
{{\Delta {\rm N}}}{\mathrm{a}}_{\mathrm{p}{\mathrm{,}}{{\mathrm{corr}}}}= \Delta {\mathrm{N}}{{\mathrm{a}}_{\mathrm{p}}}{\mathrm{ + (}}{{{\lambda }}_{{\mathrm{Na}}}}{\mathrm{/}}{{\mathrm{\lambda }}_{\mathrm{k}}}) {\mathrm{*\Delta }} {\mathrm{K}}_{\mathrm{p}}
\end{eqnarray*}

where ${\mathrm{\Delta N}}{{\mathrm{a}}_{\mathrm{p}}}$ represents the uncorrected plasma sodium change during dialysis (equivalent to ${\mathrm{\Delta }}{{\mathrm{J}}_{{\mathrm{diff}}}}$ in Equation 1), and ${\mathrm{\Delta }}{{\mathrm{K}}_{\mathrm{p}}}$ represents plasma potassium change. The ratio ${{\mathrm{\lambda }}_{{\mathrm{Na}}}}{\mathrm{/}}{{\mathrm{\lambda }}_{\mathrm{k}}}$ reflects the contributions of sodium and potassium to conductivity.

The potassium change ${\mathrm{\Delta }}{{\mathrm{K}}_{\mathrm{p}}}$ was estimated using a single-pool kinetic model based on factors such as dialysance, the volume of potassium distribution (assumed to match urea distribution), dialysis session duration, and initial blood potassium concentration (Equation 3 in Supplemental Fig. 1). Since the initial potassium level was unknown it was set at 4.8 mmol/l, the mean value in the study [1].

By deriving ${\mathrm{\Delta N}}{{\mathrm{a}}_{\mathrm{p}}}$ from Equation (1), it becomes possible to calculate the potassium-adjusted dialysate outlet sodium concentration ${\mathrm{C}}{{\mathrm{d}}_{{\mathrm{out}}}}$. This adjusted value is then used as a target to be matched by ${\mathrm{C}}{{\mathrm{d}}_{\mathrm{i}}}$.

This approach presents a problem for patients who begin dialysis with potassium levels >6 mmol/l. In such cases, the algorithm may be biased, leading to sodium loading during the session.

To investigate this further, we analyzed the effects of sodium control across seven dialysis sessions in a patient with recurrent hyperkalemia at the start of each session (Table 1). On average, we observed a plasma sodium increase of +1.2 mmol/l. However, since sodium was measured using indirect potentiometry, this increase was underestimated. A concurrent increase of 10 g/l in protein levels, attributed to hemoconcentration from ultrafiltration, was also observed. Considering an adjustment of +0.7 mmol/l in sodium for every 10 g/l increase in protein levels, we estimate that the actual sodium increase was approximately +1.9 mmol/l [4].

**Table 1:** Variations in sodium, potassium, and protein levels during seven dialysis sessions with Na control in a patient with recurrent hyperkalemia.

	Na before (mmol/l)	Na after (mmol/l)	ΔNa (mmol/l)	K before (mmol/l)	K after (mmol/l)	Protidemia before (g/l)	Protidemia after (g/l)
Session 1	137	140	+3	7.25	3.79	68	82
Session 2	143	144	+1	6	3.57	60	69
Session 3	145	147	+2	5.38	3.65	65	71
Session 4	144	146	+2	7.62	4.09	59	75
Session 5	142	145	+3	6.78	3.91	64	71
Session 6	145	144	−1	7.01		65	80
Session 7	148	146	−2	6.07	3.68		68
Mean	143.4	144.6	+1.2	6.59	3.78	63.5	73.7

Supplemental Fig. 2 shows significant variations in blood and dialysate conductivities throughout this patient's session. This underscores a limitation of the algorithm: sodium control does not consider the patient's conductivity, as illustrated by the equations above.

In their study, Maduell *et al*. measured the initial and final plasma sodium levels using the ionic dialysance biosensor integrated into the dialysis monitor, which estimated sodium based on conductivity [2]. This approach, rather than traditional blood sampling, did not allow them to distinguish between changes in sodium and potassium during the dialysis session.

In conclusion, the use of automated dialysate sodium control systems in patients with recurrent hyperkalemia at the start of dialysis sessions warrants further study to ensure these systems do not inadvertently induce significant sodium loading.

Setting the sodium change to −1 or −2 mmol/l might correct the error, but the variability in potassium levels at the start of dialysis also presents a challenge.

## Supplementary Material

sfaf018_Supplemental_File
