# Supplementary material for: Automated dialysate sodium control system: a word of caution regarding potassium
Source: Clin Kidney J. 2025 Jan 28;18(3):sfaf018. doi: 10.1093/ckj/sfaf018 (PMC11892428; doi:10.1093/ckj/sfaf018)
Supplement: sfaf018_Supplemental_File [file sfaf018_supplemental_file.docx]

**Supplemental Figure 1:**

**Equation 3: Potassium change ΔK_p_ estimated using a single-pool kinetic model**


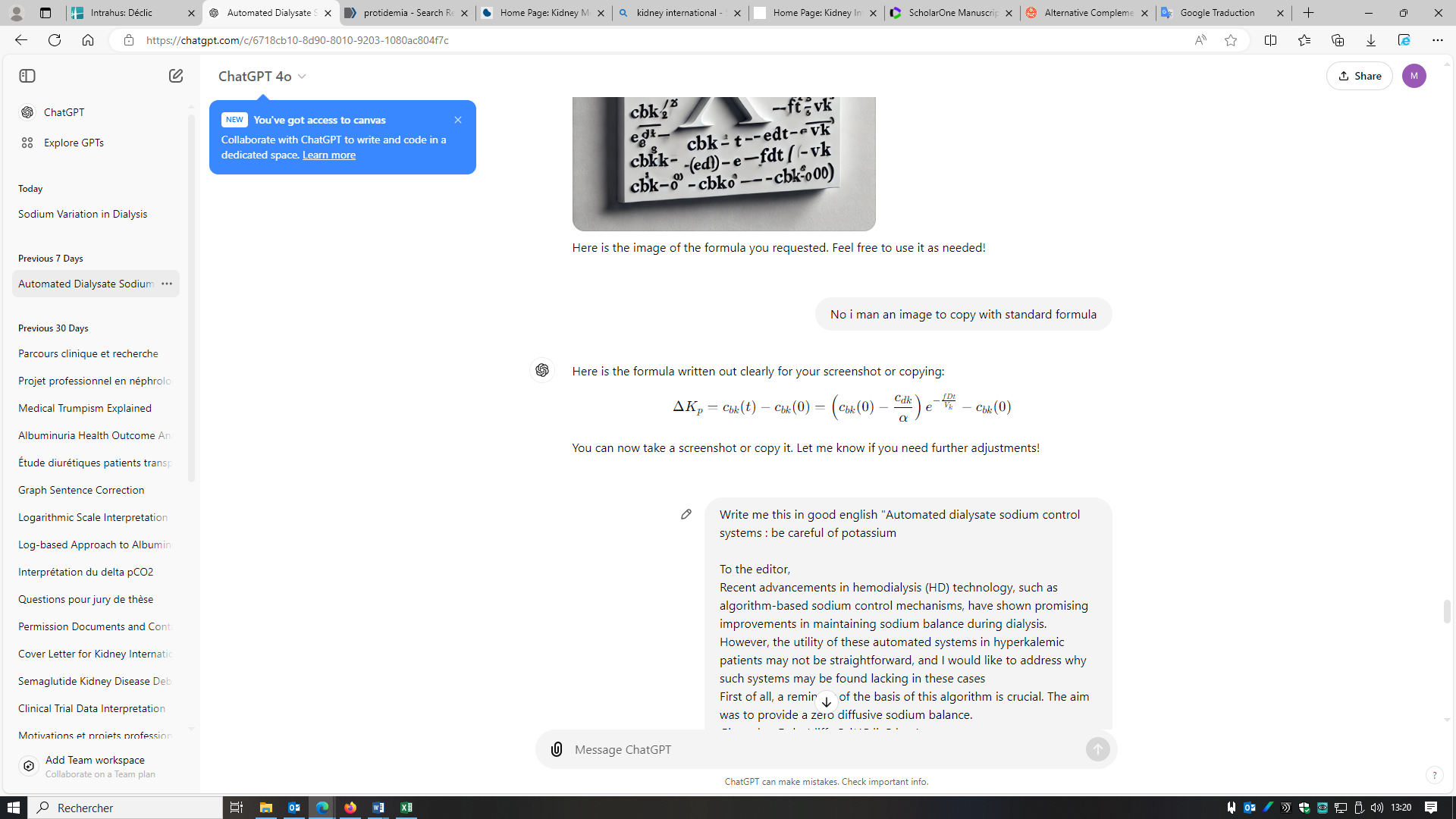


**Cbk(t) =** blood concentration in potassium at time t

**Cbk(0) =** blood concentration in potassium at time 0, arbitrarily fixed at 4.8 mmol/L

**Cdk =** dialysate concentration in potassium

**D =** Dialysance

**t =** time

**Vk =** Volume of distribution of potassium

Dt/Vk is assumed to be equal to the dialysis dose Kt/V obtained by the Online Clearance Monitor (OCM)

**f =** proportionality factor

**α =** Gibbs-Donnan factor

**Supplemental Figure 2: Instantaneous plasma and dialysate conductivity profiles during a patient’s dialysis session with hyperkalemia management and sodium control activated.**

This figure illustrates the digitized conductivity curves obtained from a dialysis session conducted with a Fresenius 6008 hemodialyzer. Data extraction was performed using WebPlotDigitizer (Ankit Rohatgi, *WebPlotDigitizer*, version 5.2, <https://automeris.io>).

The red curve represents plasma conductivity, while the blue curve depicts the conductivity of the dialysate inflow.

The figure clearly demonstrates significant fluctuations in both blood and dialysate conductivities throughout the session, underscoring the challenges faced by the algorithm in maintaining stability under these conditions.

It is important to note that plasma conductivity depends on both sodium and potassium concentrations. This may explain why plasma conductivity appears lower at the end of the dialysis session compared to the beginning.
